# Rat Paraventricular Neurons Encode Predictive and Incentive Information of Reward Cues

**DOI:** 10.3389/fnbeh.2020.565002

**Published:** 2020-09-09

**Authors:** Unur Munkhzaya, Choijiljav Chinzorig, Jumpei Matsumoto, Hiroshi Nishimaru, Taketoshi Ono, Hisao Nishijo

**Affiliations:** ^1^System Emotional Science, Faculty of Medicine, University of Toyama, Toyama, Japan; ^2^Department of Physiology, School of Bio-Medicine, Mongolian National University of Medical Sciences, Ulaanbaatar, Mongolia; ^3^Research Center for Idling Brain Science (RCIBS), University of Toyama, Toyama, Japan

**Keywords:** paraventricular nucleus of the thalamus, conditioned stimuli, predictive information, incentive information, seeking behavior

## Abstract

The paraventricular nucleus of the thalamus (PVT) has been implicated in cue-induced motivated behaviors. Although reward-associated cues (conditioned stimuli, CSs) contain different types of information including predictive information of future reward delivery and incentive (motivational) value of the reward, it remains unknown whether PVT neurons represent predictive and incentive information of CSs. It is suggested that neural activity just after the onset of CSs (early activity) and that just before reward delivery (late activity) might more strongly represent predictive and incentive information, respectively. In this study, rats were trained to lick a tube, which was protruded close to their mouth just after a CS, to obtain a reward (sucrose or water) (cue-induced licking task). Auditory and visual CSs were used: each elemental cue (CS) predicted reward or non-reward outcome, while simultaneous presentation of the two elemental cues (configural cues) predicted the opposite reward outcome. We recorded PVT neurons in the cue-induced licking task, and report that half of the CS-responsive PVT neurons responded selectively to the CSs predicting reward outcome regardless of physical property of the cues (CS^+^-selective). In addition, the early activity of the CS^+^-selective neurons discriminated reward/non-reward association (predictive information) and was less sensitive to reward value and motivation reflected by lick latency (incentive information), while the late activity of the CS^+^-selective neurons was correlated with reward value and motivation rather than reward/non-reward association. Early and late population activity of the CS^+^-selective neurons also represented predictive and incentive information of the CSs, respectively. On the other hand, activity of more than half of the PVT neurons was correlated with individual licking during licking to acquire reward. Taken together, the results suggest that the PVT neurons engage in different neural processes involved in cue-induced motivated behaviors: CS encoding to determine reward availability and form motivation for reward-seeking behavior, and hedonic mouth movements during reward consumption.

## Introduction

The paraventricular nucleus of the thalamus (PVT) is one of the midline thalamic nuclei. The PVT receives inputs from the subcortical areas related to motivation and emotion including the hypothalamus, amygdala, hippocampus, dorsal raphe, etc. (Hsu and Price, 2009; Li and Kirouac, 2012) and also inputs from the frontal cortex related to higher cognition including the anterior cingulate, prelimbic, and infralimbic cortices (Li and Kirouac, 2012). The PVT, in turn, projects to output regions for motivated behaviors (e.g., the nucleus accumbens) and emotional expression (e.g., central nucleus of the amygdala) (Penzo et al., 2015; Do-Monte et al., 2017; Dong et al., 2017). These anatomical connections of the PVT suggest that the PVT might function as an interface among the converging inputs to modulate motivational action and emotional expression (e.g., Kelley et al., 2005; Haight and Flagel, 2014).

The PVT has been implicated in reward-seeking behaviors. Presentation of cues associated with rewards (palatable food, sucrose, cocaine, ethanol, etc.) increases *c-fos* or Fos expression in the PVT (Brown et al., 1992; Dayas et al., 2008; Igelstrom et al., 2010; Choi et al., 2010; Flagel et al., 2011; James et al., 2011), and modulated Ca^2+^-fluorescent activity of PVT neurons (Choi et al., 2019; Otis et al., 2019). Lesion or inactivation of the PVT decreases reward-motivated behaviors including reward-anticipatory locomotion, locomotor sensitization to cocaine, conditioned place preference, cue- or cocaine-induced reinstatement of alcohol- or cocaine-seeking behavior, etc. (Nakahara et al., 2004; Hamlin et al., 2009; James et al., 2010; Marchant et al., 2010; Browning et al., 2014; Clark et al., 2017), while activation of the PVT neurons increases instrumental behaviors for sucrose (Labouèbe et al., 2016). These results suggest that the PVT might be involved in the transformation of information of reward-associated cues into reward-seeking motivation.

Behavioral studies suggest that reward-associated cues (conditioned stimuli, CSs) contain at least two types of information (Robinson and Berridge, 1993; Schultz, 2015); predictive information of future reward delivery and incentive (motivational) value of the reward. It has been suggested that neural activity during the initial onset of CSs (early activity) and that just before reward delivery (late activity) might more strongly represent predictive and incentive information, respectively (Fiorillo et al., 2008; Smith et al., 2011). Consistent with this idea, behavioral and neurophysiological studies reported that, when two CSs were serially presented before reward delivery, the first CS (i.e., temporally distant CS) conveyed the predictive value while the second CS (i.e., temporally proximal CS) conveyed the incentive value (Holland, 1977; Tindell et al., 2005; Meyer et al., 2014; Robinson et al., 2019). Furthermore, when a single CS was presented, the onset of CS conveyed the predictive value, while the subsequent CS period conveyed the incentive value (Ahrens et al., 2016).

Two previous neurophysiological studies reported differential neuronal responses during performance of a Pavlovian conditioning task or inhibitory responses to reward omission (Li et al., 2016; Do-Monte et al., 2017), consistent with a PVT role in motivated behaviors. However, it remains unknown how these two types of information are represented in the PVT. The present study investigated the neural representation of this information in the rat PVT by recording PVT neuronal activity in a cue-induced licking task (Oyoshi et al., 1996; Takenouchi et al., 1999; Toyomitsu et al., 2002; Matsuyama et al., 2011). The CSs consisted of both elemental (auditory or visual cues) and configural (simultaneous presentation of the auditory and visual cues) cues. In one case, each elemental CS predicted reward outcome by licking, but simultaneous presentation of those cues (configural stimulus) predicted no reward outcome. In the other case, each elemental CS predicted non-reward outcome, but simultaneous presentation of those elemental CSs (configural CS) predicted reward outcome. Here, we show that the activity of some CS-responsive PVT neurons represents predictive and incentive information of rewards regardless of stimulus sensory modality.

## Materials and Methods

### Animals

Six male Wistar rats (270–330 g; Japan SLC, Inc., Hamamatsu, Japan), were used. The rats were individually housed with free access to water and laboratory chow, where temperature was controlled at 23 ± 1°C on a 12-h light–dark cycle. The rats were treated in accordance with the policies of the National Institutes of Health on the Care of Humans and Laboratory Animals and the guidelines for experimental animals at University of Toyama. The study was approved by the Ethical Committee for Animal Experiments at University of Toyama (Permit No.: A2014MED-37 and A2017MED-16).

### Surgery

**FIGURE 1:**
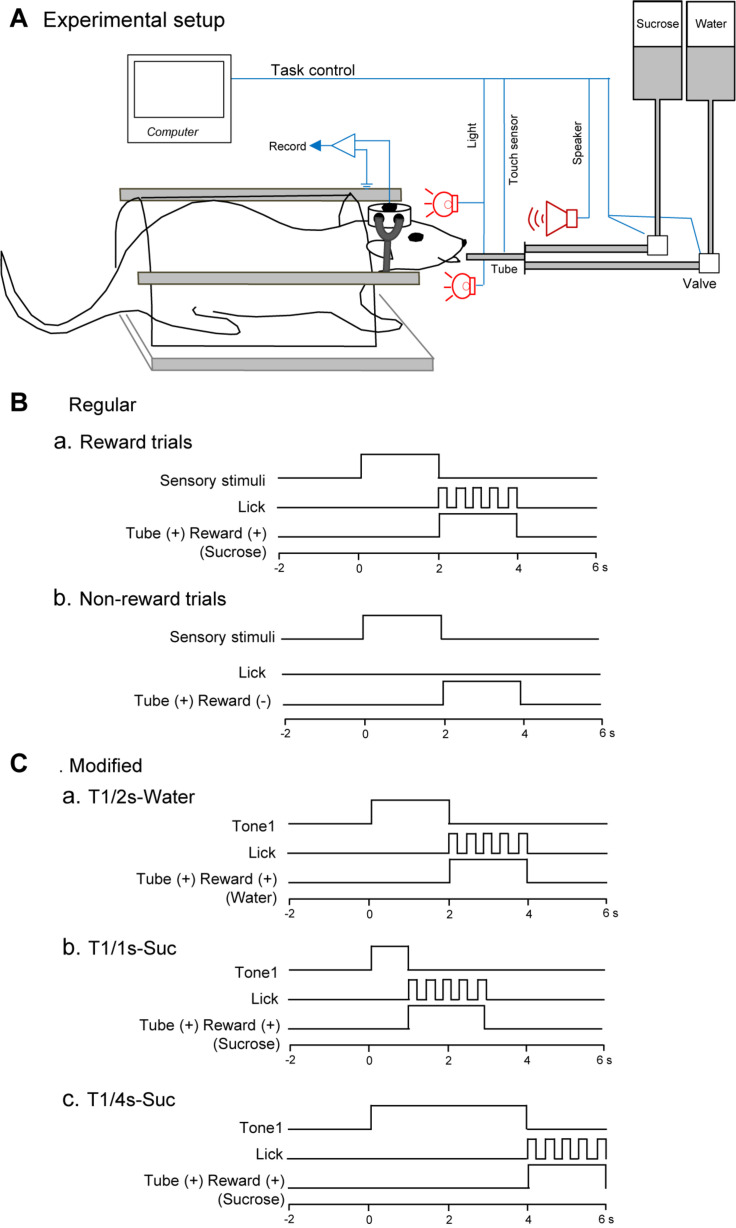
Schema of the behavioral task. **(A)** Experimental set up. Heads of the rats were painlessly fixed in a stereotaxic instrument by artificial earbars. A tube was protruded close to its mouth after the conditioned stimuli (CSs). Licking was detected by a touch sensor signaling tongue contact on the tube. **(B)** Time chart of a regular cue-induced licking task in reward **(a)** or non-reward **(b)** trials. In the reward trials **(a)**, one of the CSs (tone, light, or configural stimuli) associated with reward (sucrose) was presented for 2 s before the tube was placed close to the rat’s mouth. In the non-reward trials **(b)**, conditioned sensory stimulus and tube protrusion were similarly presented to the rat. In this situation, a reward solution was not delivered, and the rat usually did not lick the tube. **(C)** Time chart of a modified cue-induced licking task in water **(a)** and sucrose **(b,c)** trials. T1/2s-Water, 2-s Tone1 followed by water; T1/1s-Suc, 1-s Tone1 followed by sucrose; 4-s Tone1 followed by sucrose.

In accordance with our previous studies (Nishijo et al., 1998; Zou et al., 2017), the head restraint system of Nishijo and Norgren (1990, 1991, 1997) was used. After being anesthetized with an anesthetic mixture of midazolam (2 mg/kg, i.p.), medetomidine (0.15 mg/kg, i.p.), and butorphanol (2.5 mg/kg, i.p.), the acrylic dental cement was built up on the skull and small screws implanted into the skull and molded around the stainless-steel bars placed just above the skull. After the acrylic dental cement cured, these bars were removed, and an antibiotic was administered topically and systematically. These stainless-steel bars were later used as artificial earbars to painlessly hold the acrylic block on the skull in the stereotaxic instrument during a recording session (Figure 1A). Finally, a short 27-gage stainless tube, which was used as a reference pin during recording, was stereotaxically implanted in the acrylic dental cement near bregma. The coordinates of the reference pin were calibrated with reference to bregma.

After training in the cue-induced licking task (see section “Task Paradigms and Training”), a hole (diameter: 2.8–3.0 mm) was drilled through the dental cement and underlying skull (A, −1.20 to −3.6 mm from bregma; L, 0.3 mm left and right) for semi-chronic recording from the PVT under anesthesia. The dura was removed, and a few drops of antibiotics were instilled into the hole. Then, the hole was sealed with a Teflon sheet and epoxy glue. After the rat recovered (5–7 days), it was retrained in the task before recording.

### Task Paradigms and Training

Task paradigms and training were essentially similar to our previous studies (Oyoshi et al., 1996; Takenouchi et al., 1999; Toyomitsu et al., 2002; Matsuyama et al., 2011). Briefly, while the heads of the rats were painlessly fixed in a stereotaxic instrument, the rats were trained to lick a tube, which was protruded close to their mouths for 2 s after 2-s CSs, to obtain the 0.3-M sucrose solution or water (Figure 1A). The CSs consisted of auditory (2,860 or 530 Hz), visual (white light), and configural (simultaneous presentation of tone and light) stimuli (Table 1). Auditory CSs were present from a speaker 50 cm ahead of the rat, and visual CSs, from a white light in front of each eye. In reward trials of the regular cue-induced licking task, the rats licked the tube to obtain a reward (0.3 M sucrose solution or water; Figure 1Ba). A 2,860-Hz tone (Tone1), a white light in front of the right eye (Light1), or the simultaneous presentation of a 530-Hz tone and a white light in front of the left eye (Tone2 and Light2, respectively; Tone2 + Light2, configural CS) predicted reward outcome (the 0.3-M sucrose solution). In non-reward trials of the regular cue-induced licking task, Tone2, Light2, or simultaneous presentation of Tone1 and Light1 (Tone1 + Light1: configural CS) predicted no reward outcome (Figure 1Bb). In the modified cue-induced licking task, 2-s Tone1 was initially associated with water (Figure 1Ca). Then, 1 and 4-s Tone1 were associated with sucrose (Figures 1Cb,c).

**TABLE 1:** List of conditioned stimuli (CSs) used in the regular and modified cue-induced tasks.

Regular cue-induced licking task	Reward
Elemental CSs (2 s)	
Auditory CSs	
Tone1 (2,860 Hz)	Sucrose
Tone2 (530 Hz)	No reward
Visual CSs	
Light1 (right)	Sucrose
Light2 (left)	No reward
Configural CSs (2 s)	
Tone1 + Light1	No reward
Tone2 + Light2	Sucrose
**Modified cue-induced licking task**	**Reward**
Tone1 (2 s)	Water
Tone1 (1 s)	Sucrose
Tone1 (4 s)	Sucrose

The rats were initially trained with the CSs associated with and without reward in a block of 15–20 trials in each CS in the regular cue-licking task. Then, the rats were trained with the all CSs in the regular cue-induced licking task, where each CS was pseudo-randomly presented, until performance levels of the rats reached a 90–95% correct rate. Finally, the rats were trained in both regular and modified cue-induced licking tasks, as in the recording sessions (see section “Electrophysiological Recordings”). In this well-trained state, individual lick latencies to the 2-s CSs associated with sucrose were less than 300–500 ms in response to 2-s cues, consistent with previous studies (Oyoshi et al., 1996; Takenouchi et al., 1999; Toyomitsu et al., 2002; Matsuyama et al., 2011). The total number of trials per day in the training session was 200–250. A rat usually ingested 20–30 ml of liquids in the training and recording period. If the rat failed to obtain 30 ml of the liquids during the task, the remainder was given to the rat in its home cage.

### Electrophysiological Recordings

Each rat was tested every other day. After being placed in the stereotaxic instrument, a glass-insulated tungsten microelectrode (*Z* = 1.0–1.5 MΩ at 1 kHz) was stereotaxically inserted into the PVT at an angle of 10° with reference to the reference pin using a micromanipulator (SM-20, Narishige, Tokyo, Japan). The neuronal activities, CS triggers, and signals of the lick contacts on the tube were digitized and stored in a computer (MAP, Plexon Inc., Dallas, United States) system.

**FIGURE 2:**
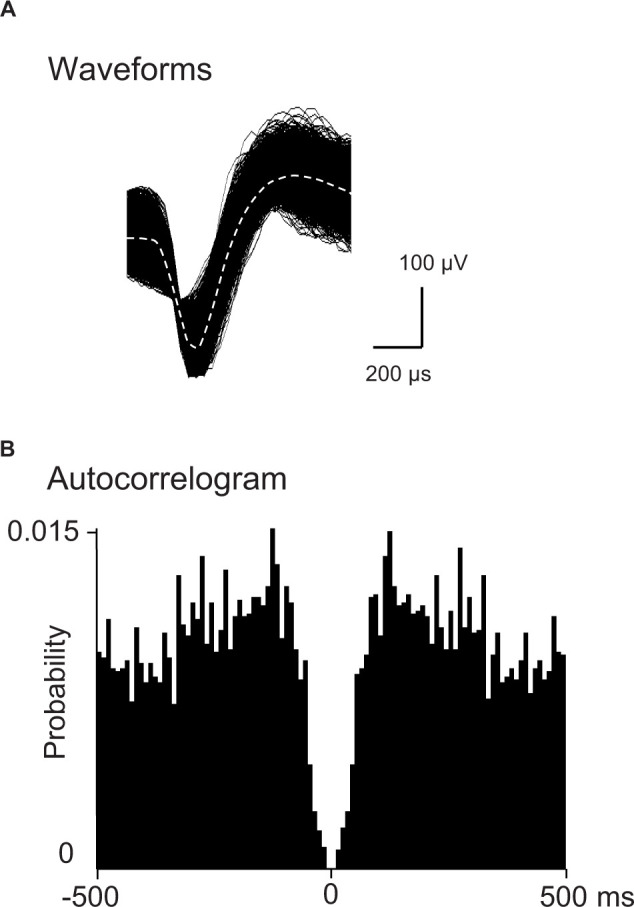
Identification of PVT neurons. **(A)** An example of superimposed waveforms of PVT neuronal activity. **(B)** Autocorrelogram of the neuronal activity shown in **(A)** The ordinate indicates probability of spikes (bin counts were divided by the number of spikes in the spike train). Bin width = 1 ms.

Spikes were isolated into single units with cluster analysis (Off-line sorter, Plexon Inc.). Then, an autocorrelogram of each unit identified by cluster analysis was analyzed: units with refractory periods≥2.0 ms in autocorrelograms were defined as single units. Furthermore, consistency of superimposed waveforms of the isolated units were inspected to confirm that the waveforms were those recorded from single units. Finally, the data were transferred to the NeuroExplorer program (Nex Technologies, Madison, AL, United States) for further analysis. Examples of superimposed waveforms of a PVT neuron are shown in Figure 2A. Figure 2B shows its autocorrelogram with the refractory period of 2 ms, suggesting that these spikes were recorded from a single unit.

When the PVT neurons had been isolated, they were initially tested with the regular cue-induced licking task (Table 1); each CS was pseudo-randomly presented with an inter-trial interval of 20–30 s, resulting in a total of 5–8 trials for each CS. Then, the neurons were sequentially tested with the modified cue-induced licking task with 8–10 trials for each condition with an inter-trial interval of 20–30 s; Tone1 (2 s) associated with water, Tone1 (1 s) associated with 0.3-M sucrose, and Tone1 (4 s) associated with 0.3-M sucrose. A previous study reported that when rats were trained to adapt to changes in reward association, the rats learned new associations within 2–3 trials (Toyomitsu et al., 2002). Therefore, in the modified cue-induced task, the data of the last five trials for each condition were analyzed and the data in the initial trials were discarded. In this modified task, water was introduced as less rewarding reinforcement compared with sucrose. Previous studies reported that manipulation, which modified motivational states, affected lick frequency (D’Aquila, 2010; Ostlund et al., 2013). Consistently, the mean lick number per trial was significantly lower in the reward trials with water than those with sucrose (Bonferroni test, *p* < 0.0001 after a one-way ANOVA) (Supplementary Figure 1). Furthermore, the 4-s CS (4-s Tone1) was tested after the 1-s CS to introduce “frustration effect” that promotes behavioral invigoration (see section “Discussion”).

### Experimental Design and Statistical Analyses

#### Data Analysis of Responses to CSs

Neuronal activity during the 2-s CS period was analyzed. The baseline firing rate was defined as the mean firing rate during the 500-ms “pre-CS” period. Significant neuronal responses (excitatory or inhibitory responses) to each CS were determined by a Wilcoxon signed rank test (*p* < 0.05) between the baseline firing rate and mean firing rate during the 2-s CS period. Response magnitude to the CS was defined as mean firing rates during the 2-s CS period minus the baseline firing rate. In each neuron, response magnitudes to all six CSs were compared by one-way ANOVAs (*p* < 0.05). PVT neurons with a significant main effect were defined as differential neurons. According to *post hoc* tests (Bonferroni test, *p* < 0.05), PVT neurons were defined as CS^+^-selective neurons if their response magnitudes to all CSs associated with reward in the regular cue-induced licking task (Tone1, Light1, and Tone2 + Light2) were larger than those to all non-rewarding CSs (Tone2, Light2, and Tone1 + Light1).

To investigate temporal characteristics of the CS^+^-related neurons, response magnitudes during the initial 500 ms after CS onset (early data set) and those during the last 500 ms of the CS (late data set) were separately analyzed. Response magnitudes in each data set were similarly defined: mean firing rates during the initial or last 500 ms of the CS period minus the baseline firing rate. These data were similarly analyzed by one-way ANOVAs (*p* < 0.05) with *post hoc* tests (Bonferroni test, *p* < 0.05). PVT neurons in the early data set were defined as early CS^+^-selective neurons if their response magnitudes to all rewarding CSs associated with reward (Tone1, Light1, and Tone2 + Light2) were larger than those to all non-rewarding CSs (Tone2, Light2, and Tone1 + Light1) (Bonferroni test, *p* < 0.05 after a one-way ANOVA). Late CS^+^-selective neurons were similarly defined based on the late data set.

Accumulating evidence suggests that neural population activity patterns represent stimulus relationships (e.g., Sereno and Lehky, 2011; Stokes et al., 2013; Chinzorig et al., 2020). Multidimensional scaling (MDS) has been widely used to decode neural population activity patterns into stimulus relationships. We hypothesized that population activity patterns of early and late CS^+^-selective neurons differently represent CS relationships: population activity patterns of early CS^+^-selective neurons might represent CS relationships based on predictive value while population activity patterns of late CS^+^-selective neurons might represent CS relationships based on incentive value. To analyze CS relationships, response magnitudes of early and late CS^+^-selective neurons were further analyzed by MDS. Each of these neurons was repeatedly (i.e., five times) tested with nine CSs (i.e., six and three CSs in the regular and modified cue-induced licking tasks, respectively), which yielded 45 stimulus arrays. Thus, the data matrices of neural activity in the 20 × 45 array derived from the 20 early CS^+^-selective neurons and that derived from the 20 late CS^+^-selective neurons were separately analyzed by MDS. In each data set, Euclidean distances (dissimilarities) between all possible pairs of CSs were calculated using the response magnitudes of each PVT neurons to the two CSs. The MDS program (PROXSCAL procedure, SPSS ver16; IBM Corporation, New York, NY, United States) positioned the CSs in a Euclidean stimulus space so that the spatial relationships among the CSs in the space represented the original relationships of the dissimilarities (Shepard, 1962). The clusters of the CSs were analyzed using the multiple discriminant analysis.

#### Data Analysis of Responses to Rewards

Single neuronal activity in response to rewards (unconditioned stimulus, US) (sucrose or water delivery for 2 s) was similarly analyzed. Significant neuronal responses (excitatory or inhibitory responses) to US after each rewarding CS were determined by a Wilcoxon signed rank test (*p* < 0.05) between the baseline firing rate and mean firing rate during the 2-s tube protrusion periods. The activity of US-responsive neurons was further analyzed to investigate neural correlation to individual licking. First, perievent histograms of neuronal spikes aligned with licking signals detected by the touch sensor (range = −80 ms to +80 ms; bin width = 20 ms) were computed using the data of the all reward trials. Then, the modulation index (MI; a normalized entropy measure; Tort et al., 2008) of the histogram was calculated, as follows:

$$\text{H}\left(\text{entropy}\right)=-\sum_{j=1}^{N}p_{j}\log p_{j}$$

$$p_{j}=\frac{C_{j}}{C}$$

$$\text{MI}=\frac{H_{max}-H}{H_{max}}$$

where *N* is the number of bins (*n* = 8), *C* is the total spike count in the histogram, *C*_j_ is the number of spike counts in the *j*^th^ bin, and *H*_max_ is the maximum possible entropy value (logN). Finally, the statistical significance of an MI value of a given US-responsive neuron was calculated by comparing it with a distribution of 200 surrogate MI values (Tort et al., 2008). The surrogate MI values were obtained by applying MI measure to trial shuffled data. The *p*-value was calculated assuming a normal distribution of the surrogate MI values. The neurons showing *p* < 0.01 and *C* > 10 were defined as neurons with significant lick correlation.

### Histological Analysis

After the all recording sessions, small electrolytic lesions (20 μA for 20 s) were stereotaxically made around the recording sites under deep anesthesia (sodium pentobarbital 100 mg/kg, i.p.). Then, rats were transcardially perfused with saline and 10% buffered formalin. The brains of the rats were cut into 50-μm frontal sections, which were stained with cresyl violet. After all lesion sites being verified under a microscope, recording sites of neurons were stereotaxically plotted on the actual brain sections. Finally, these recording sites were transferred to corresponding locations on the corresponding sections of the rat brain atlas (Paxinos and Watson, 2017). In the present study, the rat PVT was divided into its anterior (AP −1.20 to −2.40) and posterior (AP −2.40 to −3.60) parts.

Initial results were presented as an abstract at a meeting (Munkhzaya et al., 2019) and summary of a doctoral thesis (Munkhzaya, 2019).

## Results

### Responses to the CSs

**FIGURE 3:**
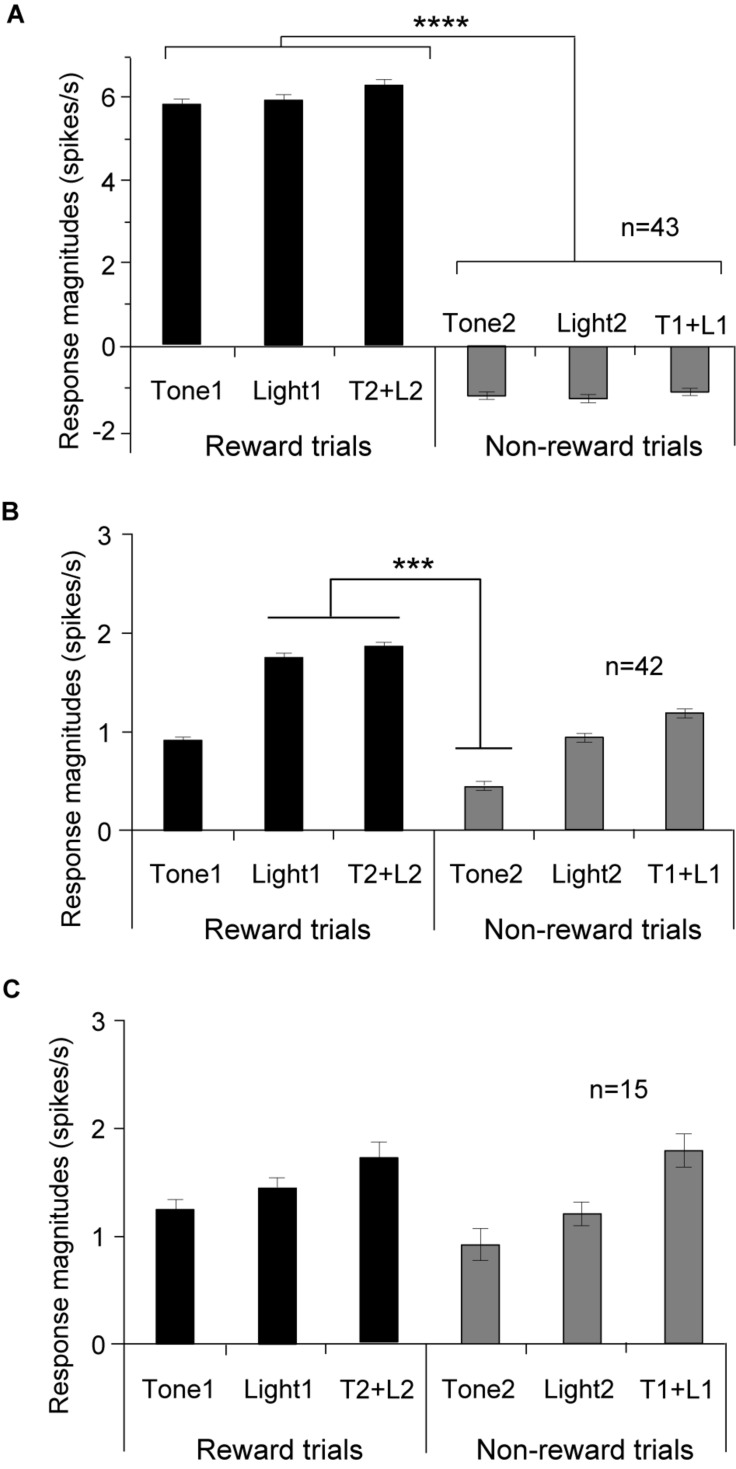
Comparison of the mean response magnitudes (mean firing rates ± standard error of the mean, SEM) to the CSs in the three subcategories of the CS-responsive neurons. **(A)** CS^+^-selective neurons (*n* = 43). Response magnitudes to the rewarding CSs are significantly greater than those to the non-rewarding CSs. **(B)** Other differential CS-responsive neurons (*n* = 42). **(C)** Non-differential CS-responsive neurons (*n* = 15). ****p < 0.0001; ***p < 0.001 (Bonferroni tests).

Of 217 PVT neurons, 100 (46.1%) showed excitatory responses to one or more CSs of the task (CS-responsive neurons). The five of these 100 neurons showed not only excitatory but also inhibitory responses, and were classified as other differential CS-responsive neurons (see below). Table 2 shows summary of the response patterns of these 100 PVT neurons. Of the 100 CS-responsive neurons, 85 (39.2%, 85/217) responded differentially to the CSs with or without reward (differential CS-responsive neurons), and 15 (6.9%) responded non-differentially (non-differential CS-responsive neurons). Of the 85 differential CS-responsive neurons, 43 (19.8%, 43/217) responded stronger to any rewarding CS than any non-rewarding CS regardless of stimulus sensory modality (CS^+^-selective neurons). Figure 3A shows the mean response magnitudes of the 43 CS^+^-selective neurons to all CSs for 2 s in the regular cue-induced licking task. A statistical analysis indicated a significant main effect of cue type [*F*(5, 252) = 33.6, *p* = 0.001]. *Post hoc* tests indicated that these neurons significantly responded stronger to the rewarding CSs (Tone1, Light1, and Tone2 + Light2) than the non-rewarding CSs (Tone2, Light2, and Tone1 + Light1) (Bonferroni test, *p* < 0.0001; see Supplementary Table 1 for individual comparisons). The remaining 42 (19.4%, 42/217) neurons differentially responded to various CSs (other differential CS-responsive neurons). These neurons responded differentially to specific CSs, but their response patterns did not match the criteria for CS^+^-selective neurons. Figure 3B shows the mean response magnitudes of the 42 other differential CS-responsive neurons to all CSs for 2 s in the regular cue-induced licking task. A statistical analysis indicated a significant main effect of cue type [*F*(5, 240) = 5.1, *p* = 0.0002]. *Post hoc* tests indicated that these neurons significantly responded stronger to Light1 and Tone2 + Light2 than Tone2 (Bonferroni test, *p* < 0.001). Figure 3C shows the mean response magnitudes of the 15 non-differential CS-responsive neurons to all CSs for 2 s in the regular cue-induced licking task. A statistical analysis indicated no significant main effect of cue type [*F*(5, 84) = 0.424, *p* = 0.831].

**TABLE 2:** Categories and numbers of the rat PVT neurons.

Classification	*n*
Responses to CSs (CS-responsive)	100
Differential CS-responsive	85
CS^+^-selective	43
Early CS^+^-selective	20
Late CS^+^-selective	20
Both early and late CS^+^-selective	8
Other CS^+^-selective	11
Other differential CS-responsive	42
**Non-differential CS-responsive**	**15**
Response to US (US-responsive)	133
Responsive only to US	59
Responsive to both CSs and US	74
Non-responsive	58
Total	217

**FIGURE 4:**
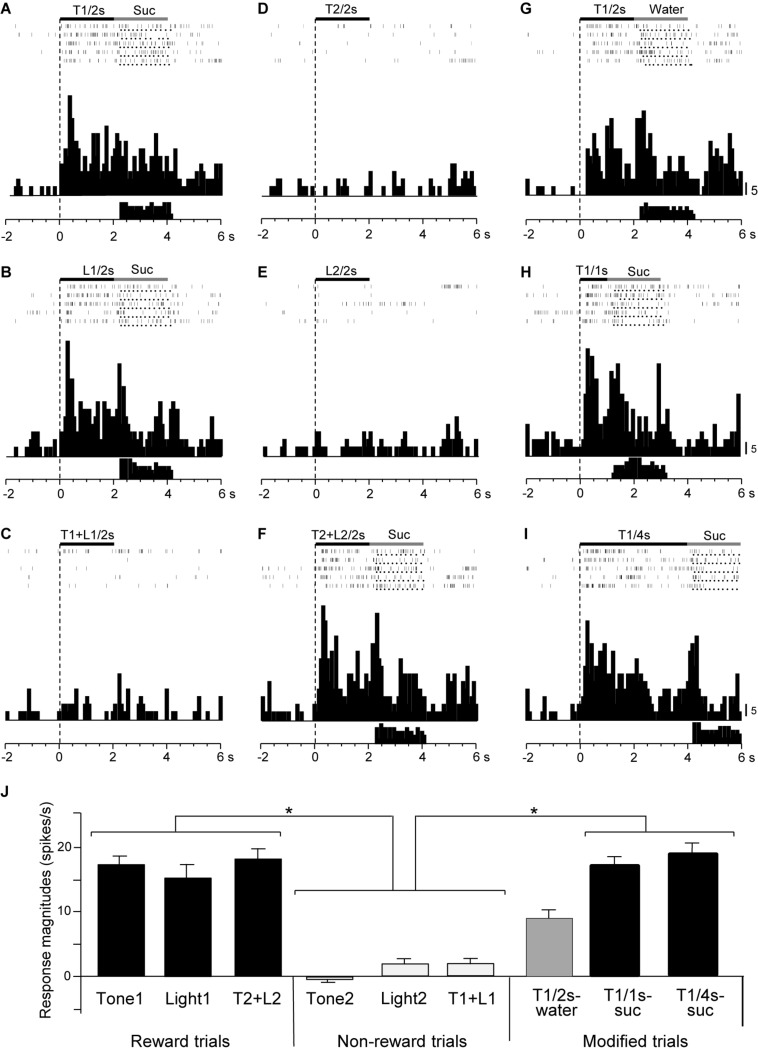
Activity of an early CS^+^-selective neuron that responds differently to CSs associated with reward and non-reward. **(A–F)** Responses to CSs in the regular cue-induced licking task. Raster displays and summed histograms indicate neuronal responses to Tone1 associated with the sucrose solution **(A)**, Light1 associated with the sucrose solution **(B)**, Tone1 + Light1 associated with non-reward **(C)**, Tone2 associated with non-reward **(D)**, Light2 associated with non-reward **(E)**, and Tone2 + Light2 associated with the sucrose solution **(F)**. **(G–I)** Responses to Tone1 associated with reward in the modified cue-induced licking task. Note that the neuron responded to the CSs associated with reward. Black and gray rectangles above the raster displays indicate CS duration and time of reward, respectively. Each dot below the raster line indicates one lick; each upper histogram shows summed neuronal responses; and each lower histogram shows summed licks. Abscissas indicate time; onset of CS at time 0; negative values represent the pre-trial control. Histogram bin width, 100 ms. Suc, 0.3 M sucrose solution. **(J)** Comparison of neuronal responses to the CSs (mean firing rate ± SEM). T1/2s, 2-s Tone1; L1/2s, 2-s Light1; T1 + L1/2s, 2-s Tone1 + Light1; T2/2s, 2-s Tone2; L2/2s, 2-s Light2; T2 + L2/2s, 2-s Tone2 + Light2; T1/1s, 1-s Tone1; T1/4s, 4-s Tone1; T1 + L1, Tone1 + Light1; T2 + L2, Tone2 + Light2; T1/2s-water, 2-s Tone1 associated with water; T1/1s-suc, 1-s Tone1 associated with sucrose; T1/4s-suc, 4-s Tone1 associated with sucrose. *p < 0.05 (Bonferroni test).

**FIGURE 5:**
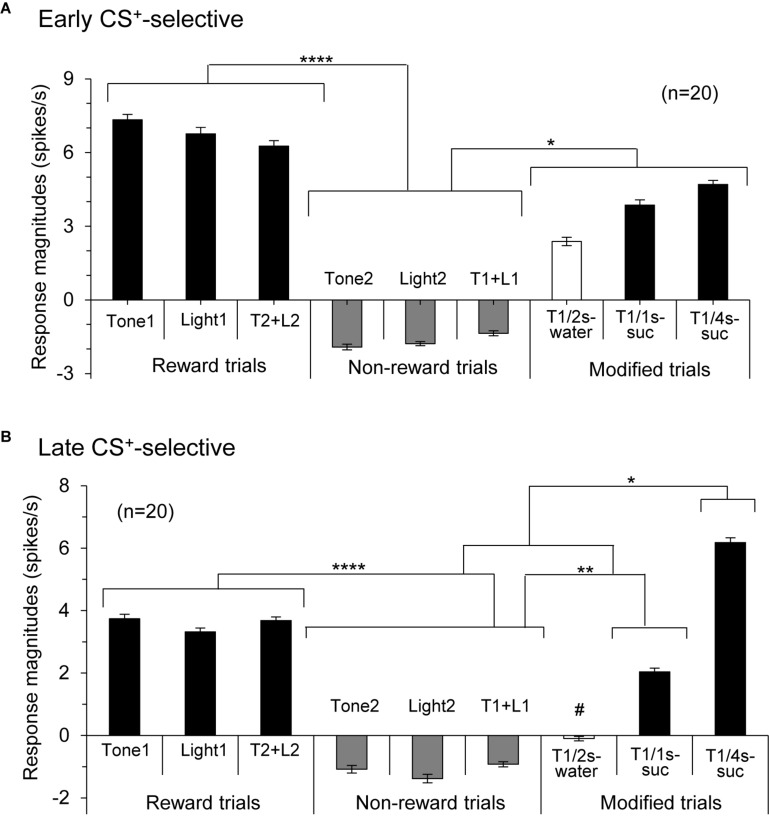
Histograms of mean response magnitudes to CSs in the early **(A)** and late **(B)** CS^+^-selective neurons (mean firing rates ± SEM). T1 + L1, Tone1 + Light1; T2 + L2, Tone2 + Light2; T1/2s-water, 2-s Tone1 associated with water; T1/1s-suc, 1-s Tone1 associated with sucrose; T1/4s-suc, 4-s Tone1 associated with sucrose. *, **, ****p < 0.05, 0.01, and 0.0001, respectively (Bonferroni test after one-way ANOVA). #, significant difference from Tone1, Light1, T2 + L2, and T1/4s-suc (*p* < 0.001).

Responses of CS^+^-selective neurons during the early and late 500 ms of the CSs were further analyzed. Of the 43 CS^+^-selective neurons, 20 and 20 neurons showed early and late CS^+^-selective responses, respectively. Of these 43 neurons, eight showed both early and late CS^+^-selective responses (Table 2 and Supplementary Figure 2). Furthermore, we analyzed neuronal activity during the middle part of the CSs (i.e., from 0.75 to 1.25 s after CS onset). However, the all 43 CS^+^-selective neurons showed no CS^+^-selective responses during the middle part of the CSs. In addition, we also analyzed neuronal activity during the early and late 500 ms of the CSs regardless of responses during the 2-s CS period, and found that another 11 neurons responded to some CSs during the early 500 ms of the CSs while another 7 neurons responded to some CSs during the late 500 ms of the CSs. However, all of these neurons showed non-differential responses to the CSs. Figure 4 shows the activity of an early CS^+^-selective neuron in the PVT. The neuron displayed excitatory responses to Tone1 (Figure 4A), Light1 (Figure 4B) and configural stimulus (Tone2 + Light2, Figure 4F) associated with sucrose solution in the regular cue-induced licking task. However, the neuron did not respond to Tone2 (Figure 4D), Light2 (Figure 4E), or configural stimulus (Tone1 + Light1, Figure 4C) predicting non-reward. Furthermore, the neuron responded to Tone1 associated with rewards (water or sucrose) in the modified cue-induced licking task (Figures 4G–I). The mean response magnitudes during the early 500 ms of the CSs are indicated in Figure 4J. The statistical analysis indicated a significant main effect of cue type [one-way ANOVA: *F*(8, 36) = 7.38, *p* = 0.001]. *Post hoc* tests revealed that response magnitudes to all CSs associated with sucrose were greater than those associated with non-reward (Bonferroni test, *p* < 0.05; see Supplementary Table 2 for individual comparisons). Figure 5A shows for the 20 early CS^+^-selective neurons their mean response magnitudes to the early 500 ms of the CSs. A statistical analysis demonstrated a significant main effect of cue type [one-way ANOVA: *F*(8, 171) = 22.5, *p* = 0.0001]. *Post hoc* tests indicated that response magnitudes to all CSs associated with rewards were greater than those associated with non-reward (Bonferroni test, *p* < 0.05; see Supplementary Table 3 for individual comparisons).

**FIGURE 6:**
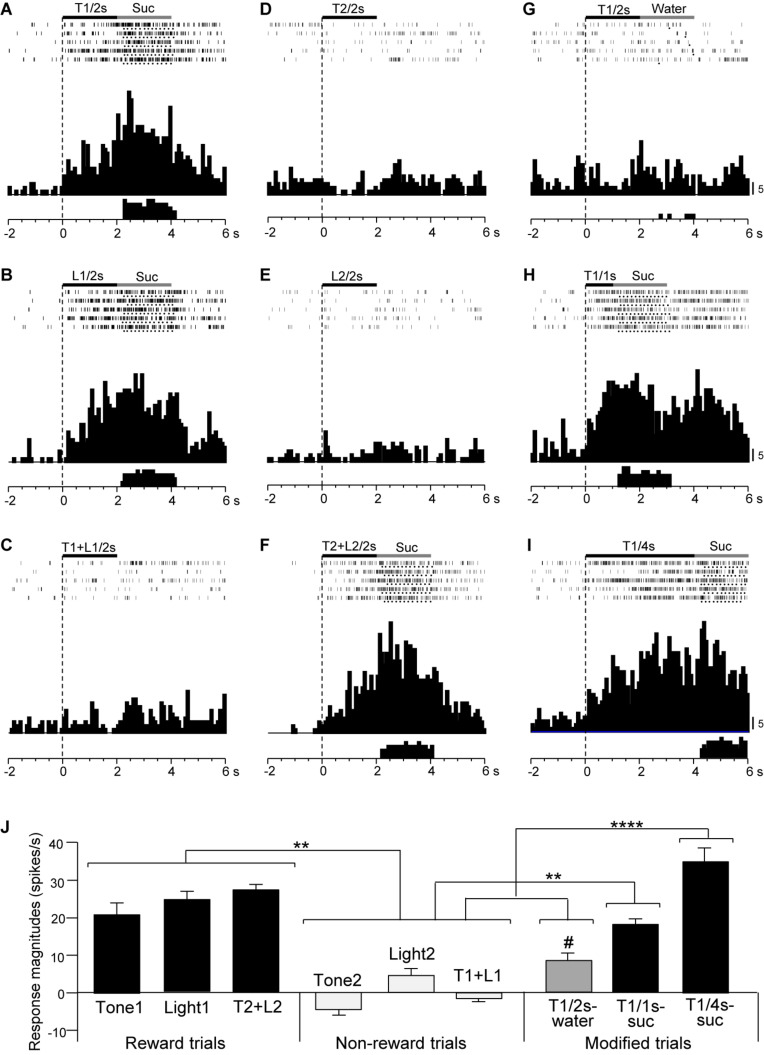
Activity of a late CS^+^-selective neuron that responded differently to CSs associated with reward and non-reward. **(A–F)** Responses to CSs in the regular cue-induced licking task. Raster displays and summed histograms indicate neuronal responses to Tone1 associated with the sucrose solution **(A)**, Light1 associated with the sucrose solution **(B)**, Tone1 + Light1 associated with non-reward **(C)**, Tone2 associated with non-reward **(D)**, Light2 associated with non-reward **(E)**, and Tone2 + Light2 associated with the sucrose solution **(F)**. **(G–I)** Responses to Tone1 associated with reward in the modified cue-induced task. Note that the neuron responded to the CSs associated with reward. **(J)** Comparison of neuronal responses to the CSs (mean firing rate ± SEM). T1/2s, 2-s Tone1; L1/2s, 2-s Light1; T1 + L1/2s, 2-s Tone1 + Light1; T2/2s, 2-s Tone2; L2/2s, 2-s Light2; T2 + L2/2s, 2-s Tone2 + Light2; T1/1s, 1-s Tone1; T1/4s, 4-s Tone1; T1 + L1, Tone1 + Light1; T2 + L2, Tone2 + Light2; T1/2s-water, 2-s Tone1 associated with water; T1/1s-suc, 1-s Tone1 associated with sucrose; T1/4s-suc, 4-s Tone1 associated with sucrose. **, ****p < 0.01 and 0.0001, respectively (Bonferroni test). #, significant difference from Tone1, Light1, and T2 + L2 (*p* < 0.05). Other descriptions as in Figure 4.

Figure 6 shows the activity of a late CS^+^-selective neuron in the PVT. The neuron displayed CS^+^-selective responses similar to those of the early CS^+^-selective neurons; excitatory responses to Tone1 (Figure 6A), Light1 (Figure 6B), and configural stimulus (Tone2 + Light2, Figure 6F) associated with sucrose solution in the regular cue-induced licking task, and those to Tone1 associated with reward (sucrose) in the modified task (Figures 6H,I). The mean response magnitudes during the late 500 ms of the CSs are shown in Figure 6J. A statistical analysis indicated a significant main effect of cue type [one-way ANOVA: *F*(8, 36) = 21.32, *p* = 0.0001]. *Post hoc* tests indicated that response magnitudes to all CSs associated with sucrose were greater than those associated with non-reward (Bonferroni test, *p* < 0.05; see Supplementary Table 4 for individual comparisons) and that the response magnitude to Tone1 (4 s) associated with sucrose (T1/4 s-suc) was greater than that to Tone1 (2 s) associated with water (T1/2 s-water) (Bonferroni test, *p* < 0.05). Figure 5B shows the mean response magnitudes of the 20 late rewarding CS^+^-selective neurons to the late 500 ms of the CSs. A statistical analysis revealed a significant main effect of cue type [one-way ANOVA: *F*(8, 171) = 24.62, *p* = 0.001]. *Post hoc* tests indicated that the response magnitudes to all CSs associated with sucrose were greater than those associated with non-reward (Bonferroni test, *p* < 0.05; see Supplementary Table 5 for individual comparisons). Furthermore, the response magnitudes to Tone1 (4 s) associated with sucrose (T1/4 s-suc) were larger than those to 2 and 1-s CSs associated with sucrose (Bonferroni test, *p* < 0.05) as well as those to Tone1 (2 s) associated with water (T1/2 s-water) (Bonferroni test, *p* < 0.0001) (see Supplementary Table 5 for individual comparisons). In addition, response magnitudes to 4- and 2-s CSs associated with sucrose were larger than those to Tone1 (2 s) associated water (T1/2 s-water) (Bonferroni test, *p* < 0.0001). Thus, response magnitudes to CSs associated with rewards tended to be larger in the following order; 4-s CS associated with sucrose >2 and 1-s CSs associated with sucrose >2-s CS associated with water.

### Relationships to Lick Latencies

**FIGURE 7:**
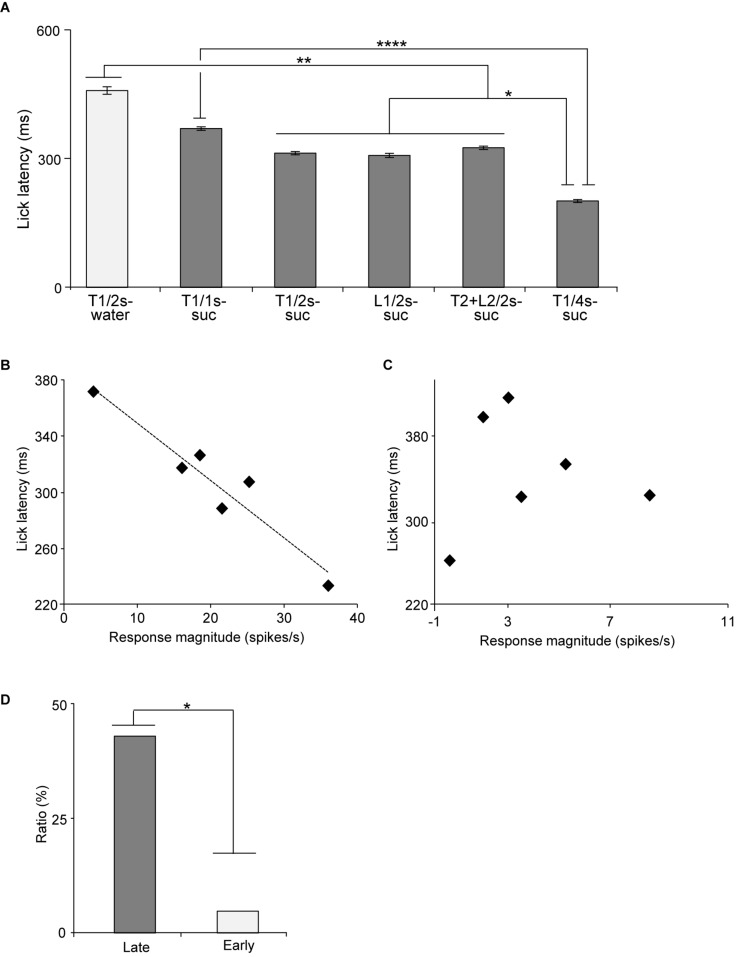
Relationships between mean lick latencies and mean response magnitudes to the CSs associated with reward in the early and late CS^+^-selective neurons. **(A)** Mean lick latencies after offset of the CSs associated with reward during recording of the late CS^+^-selective neurons (*n* = 20). **(B)** An example of a late CS^+^-selective neuron showing a significant negative correlation between mean lick latencies and mean response magnitudes to the rewarding CSs (*p* = 0.003, simple linear regression). **(C)** An example of an early CS^+^-selective neuron showing no significant correlation between mean lick latencies and mean response magnitudes to the rewarding CSs (*p* = 0.926, simple linear regression). **(D)** Comparison of the ratio of neurons with significant negative correlation between late and early CS^+^-selective neurons. T1/2s-water, 2-s Tone1 associated with water; T1/1s-suc, 1-s Tone1 associated with sucrose; T1/2s-suc, 2-s Tone1 associated with sucrose; L1/2s-suc, 2-s Light1 associated with sucrose; T2 + L2/2s-suc, 2-s Tone2 + Light2 associated with sucrose; T1/4s-suc, 4-s Tone1 associated with sucrose. *, **, ****p < 0.05, 0.01, and 0.0001, respectively.

The above results suggest that the activity of late CS^+^-selective neurons might correlate with motivation (lick latency) to reward. Latency has been reported to reflect impulsive drive to promote behaviors (Bari and Robbins, 2013; Berditchevskaia et al., 2016). Figure 7A presents the mean lick latencies after the CS offset during the recording of the late CS^+^-selective neurons. A statistical analysis indicated a significant difference among the rewarding CSs [one-way ANOVA: *F*(15, 131) = 12.4, *p* = 0.0001]. The *post hoc* comparison revealed that mean latencies for water after Tone1 (2 s) (T1/2 s-water) were longer than those for sucrose after 2 and 4-s CSs (Bonferroni test, *p* < 0.01; see Supplementary Table 6 for individual comparisons) and that the mean latencies for sucrose after Tone1 (1 s) (T1/1 s-suc) were longer than those for sucrose after Tone1 (4 s) (T1/4 s-suc) (Bonferroni test, *p* < 0.0001). Then, we analyzed the relationships between the mean lick latencies shown in Figure 7A and the mean late responses shown in Figure 5B. A simple linear regression analysis indicated a significant negative correlation between the mean response magnitudes during the last 500 ms of the rewarding CSs (Figure 5B) and lick latencies (Figure 7A) [*F*(1, 5) = 18.585, *p* = 0.0125]. Thus, stronger neuronal responses were followed by shorter licking latencies. However, the same analysis of the early CS^+^-selective neurons indicated no such significant correlation between the mean response magnitudes and lick latencies [*F*(1, 5) = 1.7974, *p* = 0.2511].

We also analyzed this correlation in individual neurons. Figure 7B shows an example of a late CS^+^-selective neuron showing a negative correlation between the response magnitudes during the late 500 ms of the rewarding CSs and the mean lick latencies after the rewarding CSs. A simple linear regression analysis indicated a significant negative correlation between response magnitudes and lick latencies [*F*(1, 5) = 42.515, *p* = 0.003]. Of the 20 late CS^+^-selective neurons, 9 (9/20, 45%) showed similar significant negative correlations between neuronal response magnitudes and lick latencies (simple linear regression, *p* < 0.05; see Supplementary Table 7 for *F*- and *p*-values of individual neurons). By contrast, only one early CS^+^-selective neuron (1/20, 5%) showed a similar significant negative correlation (simple linear regression, *F*(1, 5) = 11.8346, *p* = 0.0263; see Supplementary Table 7 for *F*- and *p*-values of individual neurons). Figure 7C shows an example of an early CS^+^-selective neuron with no correlation between the response magnitudes during the late 500 ms of the rewarding CSs and the mean lick latencies after the rewarding CSs. A simple linear regression analysis indicated no significant correlation between response magnitudes and lick latencies [*F*(1, 5) = 0.0097, *p* = 0.926]. The ratios of the neurons with negative correlation were significantly higher in the group with the late CS^+^-selective neurons compared to that with the early CS^+^-selective neurons (Fisher’s exact test, *p* = 0.0319) (Figure 7D). These results indicate that the late CS^+^-selective neurons are more important to guide seeking behaviors after the rewarding CSs.

### Temporal Representation of the CSs

**FIGURE 8:**
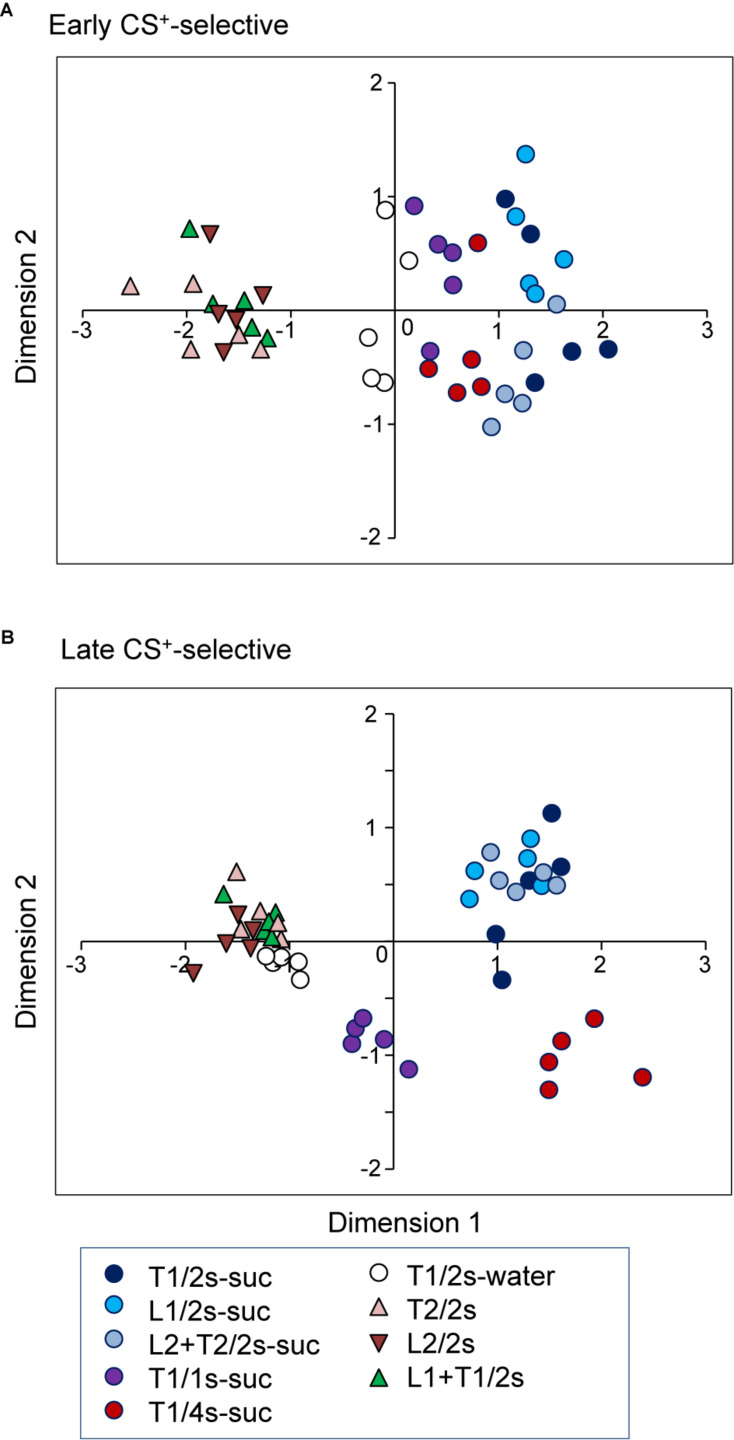
Distributions of the nine CSs in two-dimensional space resulting from multidimensional scaling (MDS) of the activities of the 20 early CS^+^-selective neurons **(A)** and the 20 late CS^+^-selective neurons **(B)**. In **(A)** three clusters were recognized (multiple discriminant analysis, *p* < 0.0001 for all analyses), while five clusters were recognized in **(B)** (multiple discriminant analysis, *p* < 0.0001). T1/2s-suc, 2-s Tone1 associated with sucrose; L1/2s-suc, 2-s Light1 associated with sucrose; L2 + T2/2s-suc, 2-s Light2 + Tone2 associated with sucrose; T1/1s-suc, 1-s Tone1 associated with sucrose; T1/4s-suc, 4-s Tone1 associated with sucrose; T1/2s-water, 2-s Tone1 associated with water; T2/2s, 2-s Tone2 associated with non-reward; L2/2s, 2-s Light2 associated with non-reward; L1 + T1/2s, 2-s Light1 + Tone1 associated with non-reward.

To investigate the temporal representation of CSs, response magnitudes to CSs in the early and late CS^+^-selective neurons were analyzed using an MDS analysis. First, the data sets of the firing rates of the 20 early CS^+^-selective neurons during the early 500 ms of the CS were subjected to an MDS analysis (Figure 8A). The *r*^2^ and stress values indicated that the stimuli were well represented in a two-dimensional space (*r*^2^ value = 0.94, stress value = 0.159). The MDS data suggest that there are three groups of CSs: CSs associated with sucrose, those with water, and those with non-reward. The multiple discriminant analysis indicated a significant separation among these three groups (Wilks’ lambda = 0.104, *p* < 0.0001).

Second, the representation of the CSs by late CS^+^-selective neuronal activity during the last 500 ms of the CS was also analyzed (Figure 8B). The *r*^2^ and stress values indicated that the stimuli were well represented in a two-dimensional space (*r*^2^ value = 0.95, stress values = 0.169). The MDS data suggest that there are five groups of CSs: CSs associated with non-rewards, 2-s CSs associated with water, 1-s CSs associated with sucrose, 2-s CSs associated with sucrose, and 4-s CSs associated with sucrose. The multiple discriminant analysis indicated a significant separation among the five groups (Wilks’ lambda = 0.006, *p* < 0.0001). It is noted that locations of CSs associated with water in the MDS space were different between the two MDS spaces derived from early and late CS^+^-selective neuronal activity. CSs associated with water were located nearer the CSs associated with non-reward in the MDS space derived from late CS^+^-selective neuronal activity compared with that derived from early CS^+^-selective neuronal activity; the mean distance between CSs associated with water and non-reward was significantly smaller in the MDS space derived from late CS^+^-selective neuronal activity (Figure 8B) than that derived from early CS^+^-selective neuronal activity (Figure 8A) [*t*-test, *t*(75) = −21.4942, *p* < 0.0001].

### Response to USs

A total of 133 neurons (61.3%, 133/217) showed excitatory responses to USs (US-responsive neurons), and did not show inhibitory responses (Table 2). Of the 133 US-responsive neurons, 59 responded only to USs, and 74 responded to both USs and CSs (Table 2). On the other hand, 11 early CS^+^-selective and 18 late CS^+^-selective neurons showed responses to USs. The ratios of US-responsive neurons were significantly greater in the late CS^+^-selective neurons (90.0%, 18/20) than the early CS^+^-selective neurons (55.0%, 11/20) (Fisher’s exact test, *p* < 0.05).

**FIGURE 9:**
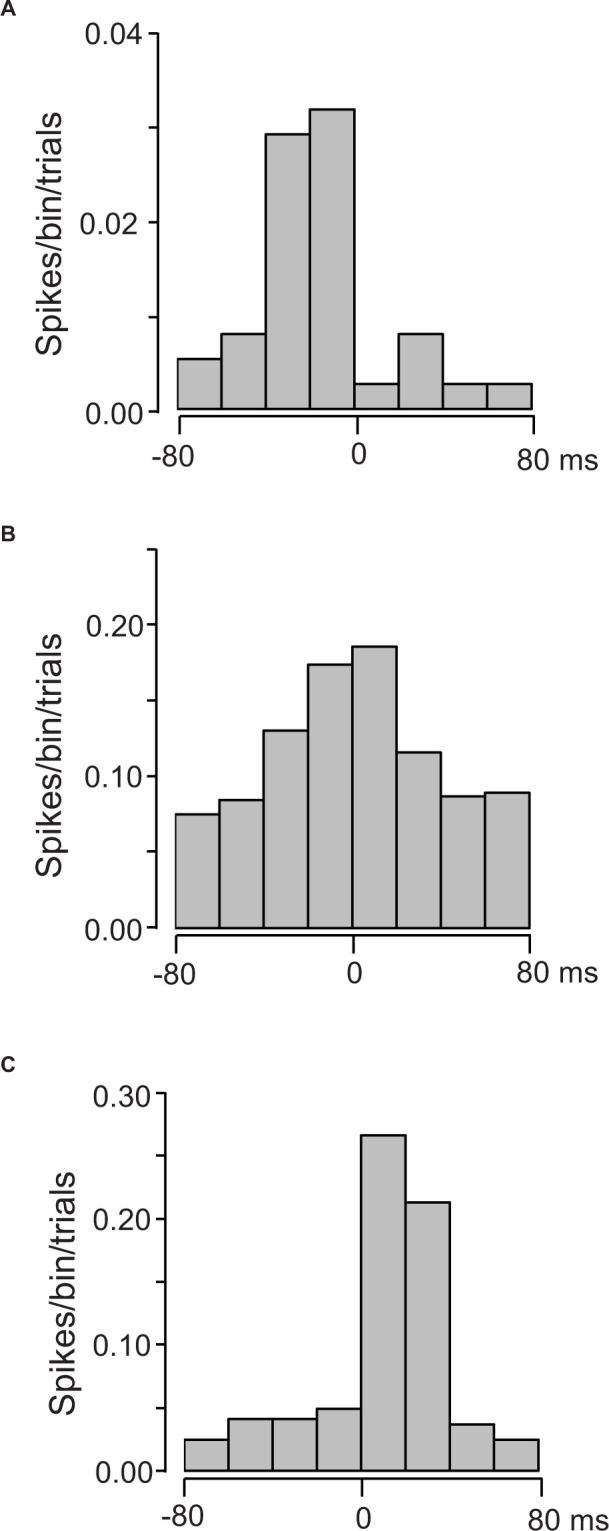
Three examples of cross-correlograms between the activity of US-responsive neurons and individual licking. **(A)** A US-responsive neuron with a peak before lick contact on the tube (MI = 0.21175, *p* < 0.0001). **(B)** A US-responsive neuron with a peak around lick contact on the tube (MI = 0.023186, *p* < 0.0001). **(C)** A US-responsive neuron with a peak after lick contact on the tube (MI = 0.021035, *p* < 0.0001). Zero on time scale indicates tongue contact with the tube. Bin width = 20 ms.

The 38 of the 133 US-responsive neurons showed a significant correlation to individual lickings. Figure 9 presents three examples of these correlations; US-responsive neurons showed activity increases before tongue contact on the tube (Figure 9A), around tongue contact (Figure 9B), and after tongue contact (Figure 9C).

### Locations of the PVT Neurons

**FIGURE 10:**
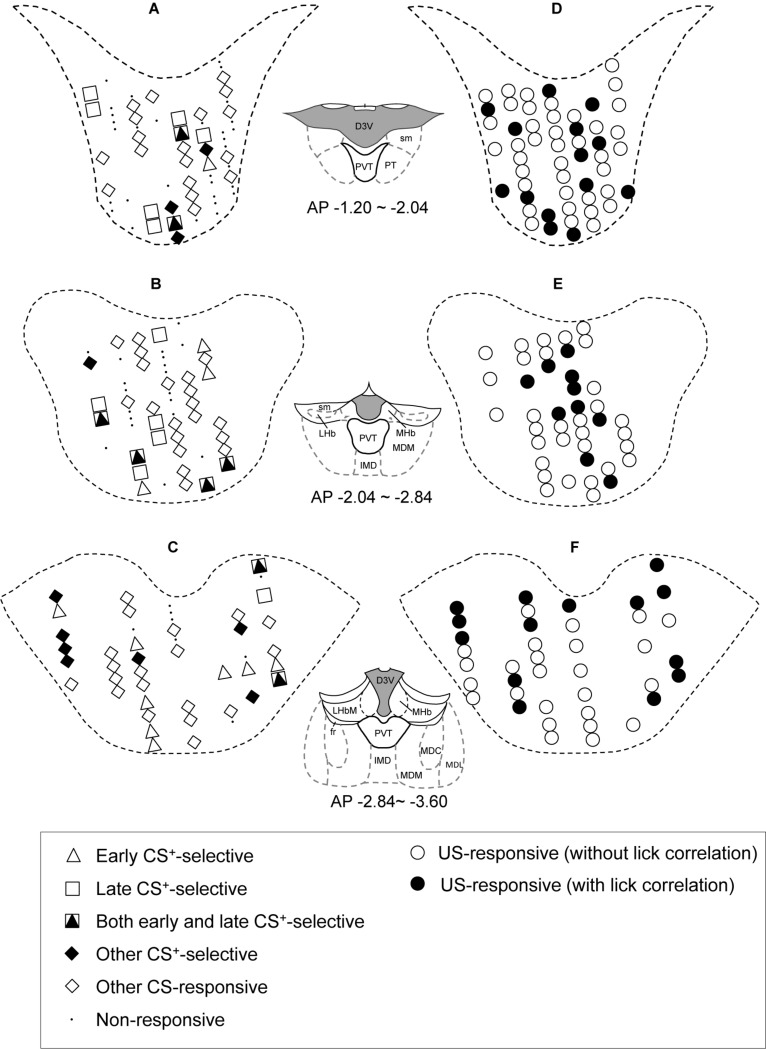
Recording sites of the PVT neurons. **(A–C)** Distributions of CS-responsive neurons. **(D–F)** Distributions of US-responsive neurons. PVT neurons are plotted on coronal sections. AP (anterior-posterior) number in each section indicates the distance (mm) posterior from the bregma. Other CS-responsive, other differential CS-responsive neurons plus non-differential CS-responsive neurons in Table 2. D3V, dorsal 3^*rd*^ ventricle; LHb, lateral habenular nucleus; sm, stria medullaris; PVT, paraventricular nucleus of the thalamus; PT, paratenial thalamic nucleus; MHb, medial habenular nucleus; MDM, mediodorsal nucleus of the thalamus, medial part; IMD, intermediodorsal nucleus of the thalamus; fr, fasciculus retroflexus; MDL, mediodorsal nucleus of the thalamus, lateral part; MDC, mediodorsal nucleus of the thalamus, central part.

**FIGURE 11:**
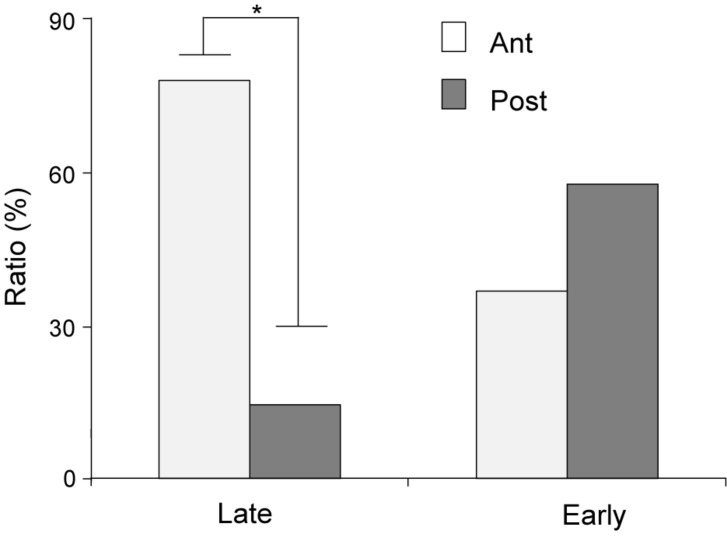
Ratios of early and late CS^+^-selective neurons in the anterior and posterior parts of the PVT. Late, late CS^+^-selective neurons; Early, early CS^+^-selective neurons; Ant, anterior part of the PVT; Post, posterior part of the PVT. *p < 0.05.

The distributions of CS- and US-responsive neurons are shown in Figures 10A–F, respectively. The late CS^+^-selective neurons (open squares and filled triangles with open squares) were located more densely in the anterior part of the PVT, while early CS^+^-selective neurons (open triangles and filled triangles with open squares) were located more evenly throughout the PVT. The ratio of the late CS^+^-selective neurons to the CS^+^-selective neurons was significantly higher in the anterior part than in the posterior part of the PVT (Fisher’s exact test, *p* = 0.0001), while there was no significant difference between these ratios for the early CS^+^-selective neurons (Fisher’s exact test, *p* = 0.227) (Figure 11). The US-responsive neurons with and without lick correlation (filled and open circles) were also located evenly throughout the PVT. There was no significant difference in the ratio of US-responsive neurons with lick correlation to all US-responsive neurons between the anterior and posterior parts of the PVT (Fisher’s exact test, *p* = 0.0858).

## Discussion

### Response Characteristics of the CS^+^-Selective PVT Neurons

The present results indicate that more than 50% of the differential CS-responsive neurons (i.e., CS^+^-selective PVT neurons, 52.9%) responded selectively to the CSs associated with reward. These differential responses to the CSs were independent of physical properties of the CSs. The CS^+^-selective PVT neurons responded selectively to the elemental CSs associated with reward regardless of physical properties of the CSs. Furthermore, these neurons also responded selectively to the configural CSs (i.e., simultaneous presentation of the auditory and visual CSs) associated with reward. It is noted that the reward predictability of the CSs in compound (i.e., configural CSs) was opposite to that of the elemental CSs presented alone, although the exact same sensory modalities were involved. These findings indicate that selective neuronal responses to the CSs are attributed to the reward predictability of the CSs rather than to the physical properties of the CSs. These results suggest strongly that the CS^+^-selective neurons are involved in the detection of cues associated with reward, consistent with a role of the PVT in cue-induced motivated behaviors (see section “Introduction”). Consistent with the present results, a presentation of cues associated with rewards (palatable food, sucrose, cocaine, ethanol, etc.) increases *c-fos* or Fos expression in the PVT (see section “Introduction”). The PVT receives afferent projections from the prefrontal cortex, amygdala, and septum (Hsu and Price, 2009; Li and Kirouac, 2012), where similar CS^+^-selective neurons have been reported (Takenouchi et al., 1999; Toyomitsu et al., 2002; Matsuyama et al., 2011). Furthermore, optogenetic manipulation of responses in prefrontal neurons projecting to the PVT suppresses conditioned reward-seeking or retrieval of conditioned cues (Do-Monte et al., 2015; Otis et al., 2017). These findings suggest that the PVT integrates information of conditioned cues from these PVT-projecting areas.

Previous studies suggest that CSs have both predictive and incentive properties (Robinson and Berridge, 1993; Robinson and Flagel, 2009; Robinson et al., 2019). The predictive property of CSs indicates the availability of rewards in the near future, while the incentive property reflects incentive motivation or salience to evoke seeking behaviors (see section “Introduction”). Especially, incentive property enhances ongoing instrumental actions (Haight and Flagel, 2014). In our study, the late CS^+^-selective neurons showed larger mean response magnitudes during the late 500 ms of the CS in the following order; 4-s CS associated with sucrose >2 and 1-s CSs associated with sucrose >CS associated with water. These differences in response magnitudes were negatively correlated with lick latencies. Analyses of the individual late CS^+^-selective neurons also indicated that 45% of the late CS^+^-selective neurons showed similar negative correlations. It is noted that the 4-s CS (Tone1) followed by sucrose was tested after 1-s CS followed by sucrose in the modified task. Introduction of the 4-s CS after the 1-s CS means reward omission at the time point 1 s after the CS onset. Previous studies reported that “frustration effect” is observed after trials with reward omission: a specific anticipated reward becomes more attractive after it has been omitted (Amsel and Roussel, 1952; Stout et al., 2003; Freidin and Mustaca, 2004). These findings suggest that behavioral and neurophysiological changes in response to the 4-s CS are attributed to frustration effect. Consistent with this idea, a previous study reported that uncertain association of reward with CSs enhanced attraction to a temporally proximal CS that conveyed incentive value (Robinson et al., 2019). Furthermore, late responses might also reflect motor preparation process that might reduce lick latencies. Human behavioral and EEG studies reported that reward affects this process (Mir et al., 2011; Schevernels et al., 2014), which might be mediated through the basal ganglia including the ventral striatum (Pasquereau et al., 2007; Galaro et al., 2019). The PVT might affect lick latency through its projections to the nucleus accumbens (see below). By contrast, the early CS^+^-selective neurons showed no significant differences in mean response magnitudes during the initial 500 ms of the CS among the rewarding CSs, although response magnitudes to the CSs associated with rewards were greater than those to CSs associated with non-reward. Furthermore, there was no significant correlation between the mean response magnitudes of the early CS^+^-selective neurons and mean lick latencies. Analyses of the individual neurons indicated that only 5% of the early CS^+^-selective neurons showed similar negative correlations. These results suggest that the activity of early CS^+^-selective neurons reflects reward/non-reward contingency of CSs, while activity of late CS^+^-selective neurons reflects the motivational significance of CSs. The MDS analyses support the above results. The MDS analysis of the early CS^+^-selective neurons indicated three clusters of the CSs: CSs associated with non-reward, water, and sucrose. The MDS analysis of the late CS^+^-selective neurons revealed five clusters of the CSs: CSs associated with non-reward, CS associated with water, 1-s CSs associated with sucrose, 2-s CSs associated with sucrose, and 4-s CS associated with sucrose, which were correlated to lick latencies. It is noted that the presentation of the CS associated with water resulted in longer latencies and that the same CSs associated with water were located nearer the CSs associated with non-reward in the MDS space based on the late CS^+^-selective neuronal activity. Taken together, these results suggest that the activity of early CS^+^-selective neurons reflects the predictive property of CSs, while activity of late CS^+^-selective neurons reflects the incentive property of CSs.

In the present study, the late CS^+^-selective neurons were more densely located in the anterior part of the PVT. Neuroanatomical studies reported that the anterior and posterior parts of the PVT have different anatomical connections; the posterior part of the PVT has stronger connections with areas related to stress and fear expression such as the amygdala (Vertes and Hoover, 2008; Li and Kirouac, 2008, 2012). Furthermore, optogenetic, genetic, or pharmacological manipulations of the anterior part of the PVT affect reward-seeking behaviors (Choi et al., 2012; Barson et al., 2015, 2017; Do-Monte et al., 2017), while *c-fos* expression increases in the anterior part of the PVT when motivation for food is increased by various behavioral and pharmacological manipulations (Warne et al., 2007; Choi et al., 2010; Mitra et al., 2011). In addition, optogenetic activation of projections from the anterior part of the PVT to the nucleus accumbens increased motivation for feeding in a stressful condition (Cheng et al., 2018), and cocaine treatment increased firing rates of neurons in the anterior part of the PVT (Yeoh et al., 2014). These findings suggest that the anterior part of the PVT controls motivated behaviors, which might be subserved partly by late CS^+^-selective neurons. However, it should be noted that this difference between the anterior and posterior parts of the PVT is rather quantitative in the present study since some late CS^+^-selective neurons were also located in the posterior part of the PVT, consistent with a previous study showing that genetic and anatomical characteristics of the PVT gradually changed from its anterior to posterior parts (Gao et al., 2020).

### Neural Mechanisms of Reward-Seeking Behaviors

The PVT is one of the important areas in the meso-cortico-limbic circuits involved in reward “wanting” and hedonic “liking” (Richard et al., 2013). The PVT receives cue information from the prefrontal cortex, amygdala, and septum and sends projections to the nucleus accumbens (see section “Introduction”). Projection fibers from the PVT to the nucleus accumbens are glutamatergic/aspartatergic (Christie et al., 1987; Frassoni et al., 1997), and stimulation of the PVT increases dopamine release in the nucleus accumbens (Jones et al., 1989; Pinto et al., 2003; Parsons et al., 2007). It is reported that presentation of CSs associated with reward increases glutamate release, as well as dopamine release in the nucleus accumbens (Roitman et al., 2004; Batten et al., 2018), that increases in dopamine levels in the nucleus accumbens, are associated with the occurrence of operant action in response to CSs associated with reward (Roitman et al., 2004; Ko and Wanat, 2016), and that depletion of dopamine in the nucleus accumbens delays the latency of operant responses after the onset of CSs (Cole and Robbins, 1989). Furthermore, a recent study reported that dopaminergic projections to the nucleus accumbens core and shell were involved in reward association for instrumental responses and motivation for the responses, respectively (Heymann et al., 2020). Taken together, these findings suggest that the PVT, especially the late CS+-selective neurons, might enhance reward-seeking behaviors, as indicated by lick latency, through dopamine levels in the nucleus accumbens.

Unconditioned stimulus-responsive neurons with and without lick correlation were located in both the anterior and posterior parts of the PVT. Neurons with lick correlation have been reported in the PVT although their rostro-caudal presence has not been described yet (Li et al., 2016). The PVT receives projections from the superior colliculus (Krout et al., 2001), which is involved in oral sensory information processing and control of rhythmic tongue and oral movements (Auroy et al., 1991; Moore et al., 2014). In turn, the PVT sends projections to the nucleus accumbens (Vertes and Hoover, 2008; Li and Kirouac, 2008), which is involved in hedonic rhythmic mouth and tongue movements during ingestion of a sweet reward (Richard et al., 2013). There are two types of mouth movements in rodents (Grill and Norgren, 1978; Steiner et al., 2001): sweet solutions induce rhythmic tongue movements and mouth expressions of “liking,” while bitter solutions induce “disgust” gapes. Rhythmic licking movements during ingestion of sucrose and water in the present study might correspond to “liking” expression of mouth movements. Consistent with this idea, the mean number of licking was increased during ingestion of sucrose compared to water. These findings suggest that US-responsive neurons with lick correlation might be involved in hedonic reaction in response to palatable food through its connection to the nucleus accumbens.

In conclusion, the present results suggest that the anterior and posterior parts of the PVT are organized heterogeneously and that the PVT neurons engage in different neural processes involved in a cue-induced motivated behavior: CS encoding to determine reward availability and form motivation for reward-seeking behavior, and hedonic mouth movements during reward consumption. However, it should be noted that there were no direct measures of incentive values of the CSs in the present study, since it is difficult to test animals with various behavioral tests (such as approach, conditioned reinforcement, and Pavlovian-instrumental transfer) while single PVT neuronal activity was simultaneously recorded from head-fixed animals. Instead, we measured lick latency of the animals, which has been reported to reflect impulsive drive of animals (Bari and Robbins, 2013; Berditchevskaia et al., 2016). Further studies using psychostimulants such as amphetamine, which increased neuronal responses to temporally proximal cues (Tindell et al., 2005), would be interesting to observe changes in firing rates of late CS^+^-selective neurons.

## Data Availability Statement

The raw data supporting the conclusions of this article will be made available by the authors, without undue reservation.

## Ethics Statement

The animal study was reviewed and approved by Ethical Committee for Animal Experiments at University of Toyama.

## Author Contributions

HNJ designed the experiment. UM and CC performed the experiment. UM, CC, and HNJ analyzed the data and wrote the manuscript. UM, CC, JM, HNM, TO, and HNJ revised the manuscript. All authors discussed the results and approved the final manuscript.

## Conflict of Interest

The authors declare that the research was conducted in the absence of any commercial or financial relationships that could be construed as a potential conflict of interest.
