# Supplementary material for: Rat Paraventricular Neurons Encode Predictive and Incentive Information of Reward Cues
Source: Front Behav Neurosci. 2020 Sep 9;14:565002. doi: 10.3389/fnbeh.2020.565002 (PMC7509094; doi:10.3389/fnbeh.2020.565002)
Supplement: Supplementary file 1 [file Presentation_1.pdf]

Supplementary Material

Rat paraventricular neurons encode predictive and incentive information of reward cues

Unur Munkhzaya, Choijiljav Chinzorig, Jumpei Matsumoto, Hiroshi Nishimaru, Yusaku Takamura, Taketoshi Ono, and Hisao Nishijo

*** Correspondence:** Dr. Hisao Nishijo, nishijo@med.u-toyama.ac.jp

Supplementary figures

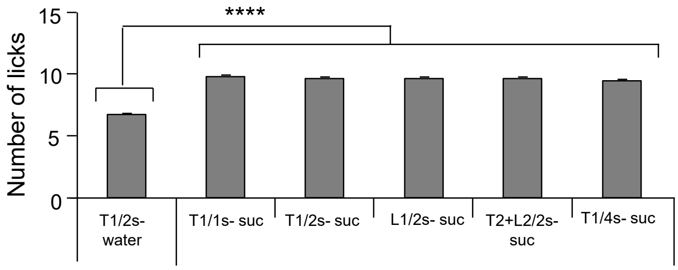

Supplementary Figure 1. Comparison of numbers of licks per trial in the reward trials (mean number of licks ± SEM). The data of lick frequency per trial were analyzed when 43 CS^+-selective neurons were recorded. The mean numbers of licks per trial during 2 s after the CSs were significantly lower in the water trials (Tone1 associated with water) than the sucrose trials (CSs associated with sucrose). T1/2s-water, water following 2-s Tone1; T1/1s-suc, sucrose following 1-s Tone1; T1/2s-suc, sucrose following 2-s Tone1; L1/2s-suc, sucrose following 2-s Light1; T2+L2/2s-suc, sucrose following 2-s Tone2+Light2; T1/4s-suc, sucrose following 4-s Tone1. ****, $p < 0.0001$ (Bonferroni test).

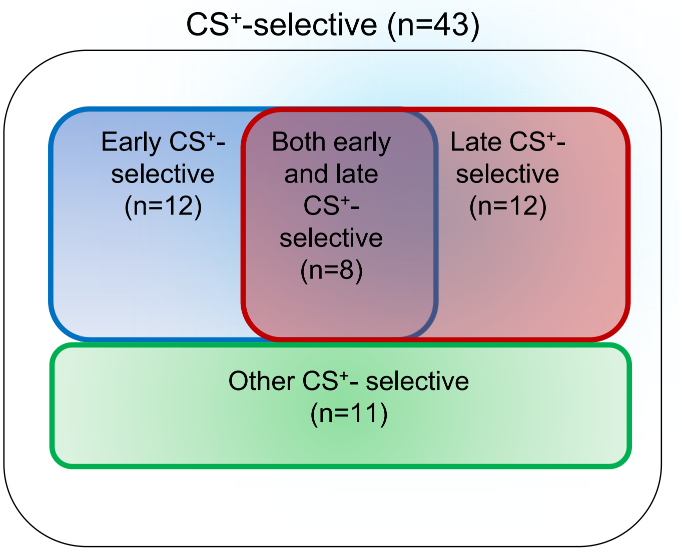

Supplementary Figure 2. A Venn diagram showing response patterns of 43 CS^{+}-selective neurons in the PVT.

Supplementary tables

Supplementary Table 1. Summary of post-hoc statistical comparisons (Bonferroni test) in Fig. 3.

Stimulus pairs	p-values
Tone1 vs. Light1	0.999999
Tone1 vs. Tone2+Light2	0.996620
Tone1 vs. Tone2	2.07E-08
Tone1 vs. Light2	2.07E-08
Tone1 vs. Tone1+Light1	2.07E-08
Light1 vs. Tone2+Light2	0.999314
Light1 vs. Tone2	2.07E-08
Light1 vs. Light2	2.07E-08
Light1 vs. Tone1+Light1	2.07E-08
Tone2+Light2 vs. Tone2	2.07E-08
Tone2+Light2 vs. Light2	2.07E-08
Tone2+Light2 vs. Tone1+Light1	2.07E-08
Tone2 vs. Light2	1.000000
Tone2 vs. Tone1+Light1	0.999997
Light2 vs. Tone1+Light1	0.999985

Supplementary Table 2. Summary of post-hoc statistical comparisons (Bonferroni test) in Fig. 4J.

**Stimulus pairs**	**p-value**
Tone1 vs. Light1	1.000000
Tone1 vs. T2+L2	0.998993
Tone1 vs. Tone2	0.014854
Tone1 vs. Light2	0.038966
Tone1 vs. T1+L1	0.038966
Tone1 vs. T1/2s-water	0.503158
Tone1 vs. T1/1s-suc	0.999985
Tone1 vs. T1/4s-suc	0.997414
Light1 vs. T2+L2	0.997414
Light1 vs. Tone2	0.019028
Light1 vs. Light2	0.048997
Light1 vs. T1+L1	0.048997
Light1 vs. Tone1/2s-water	0.564034
Light1 vs. T1/1s-suc	0.999916
Light1 vs. T1/4s-suc	0.994228
T2+L2 vs. Tone2	0.002389
T2+L2 vs. Light2	0.006911
T2+L2 vs. T1+L1	0.006911
T2+L2 vs. T1/2s-water	0.170265
T2+L2 vs. T1/1s-suc	0.999998
T2+L2 vs. T1/4s-suc	1.000000
Tone2 vs. Light2	0.999985
Tone2 vs. T1+L1	0.999985
Tone2 vs. T1/2s-water	0.742537
Tone2 vs. T1/1s-suc	0.005320
Tone2 vs. T1/4s-suc	0.001820
Light2 vs. T1+L1	1.000000
Light2 vs. T1/2s-water	0.917909
Light2 vs. T1/1s-suc	0.014854
Light2 vs. T1/4s-suc	0.005320
T1+L1 vs. T1/2s-water	0.917909
T1+L1 vs. T1/1s-suc	0.014854
T1+L1 vs. T1/4s-suc	0.005320
T1/2s-water vs. T1/1s-suc	0.286714
T1/2s-water vs. T1/4s-suc	0.140770
T1/1s-suc vs. T1/4s-suc	0.999985

Supplementary Table 3. Summary of post-hoc statistical comparisons (Bonferroni test) in Fig. 5A.

Stimulus pairs	p-value
Tone1 vs. Light1	0.999890
Tone1 vs. T2+L2	0.989803
Tone1 vs. Tone2	8.97E-08
Tone1 vs. Light2	8.97E-08
Tone1 vs. T1+L1	8.97E-08
Tone1 vs. T1/2s-water	3.58E-04
Tone1 vs. T1/1s-suc	5.18E-02
Tone1 vs. T1/4s-suc	0.316910
Light1 vs. T2+L2	0.999960
Light1 vs. Tone2	8.97E-08
Light1 vs. Light2	8.97E-08
Light1 vs. T1+L1	8.97E-08
Light1 vs. Tone1/2s-water	3.10E-03
Light1 vs. T1/1s-suc	1.94E-01
Light1 vs. T1/4s-suc	0.659221
T2+L2 vs. Tone2	8.97E-08
T2+L2 vs. Light2	8.97E-08
T2+L2 vs. T1+L1	9.01E-08
T2+L2 vs. T1/2s-water	1.61E-02
T2+L2 vs. T1/1s-suc	0.447819
T2+L2 vs. T1/4s-suc	0.902354
Tone2 vs. Light2	1.000000
Tone2 vs. T1+L1	0.999904
Tone2 vs. T1/2s-water	4.10E-03
Tone2 vs. T1/1s-suc	9.47E-06
Tone2 vs. T1/4s-suc	2.20E-07
Light2 vs. T1+L1	0.999989
Light2 vs. T1/2s-water	6.59E-03
Light2 vs. T1/1s-suc	1.81E-05
Light2 vs. T1/4s-suc	3.67E-07
T1+L1 vs. T1/2s-water	2.45E-02
T1+L1 vs. T1/1s-suc	1.17E-04
T1+L1 vs. T1/4s-suc	2.50E-06
T1/2s-water vs. T1/1s-suc	0.926377
T1/2s-water vs. T1/4s-suc	0.497070
T1/1s-suc vs. T1/4s-suc	0.998072

Supplementary Table 4. Summary of post-hoc statistical comparisons (Bonferroni test) in Fig. 6J.

**Stimulus pairs**	**p-value**
Tone1 vs. Light1	0.999376
Tone1 vs. T2+L2	0.961938
Tone1 vs. Tone2	0.000013
Tone1 vs. Light2	0.002525
Tone1 vs. T1+L1	0.000144
Tone1 vs. T1/2s-water	0.034687
Tone1 vs. T1/1s-suc	1.000000
Tone1 vs. T1/4s-suc	0.097706
Light1 vs. T2+L2	0.999800
Light1 vs. Tone2	0.000002
Light1 vs. Light2	0.000414
Light1 vs. T1+L1	0.000022
Light1 vs. Tone1/2s-water	0.006821
Light1 vs. T1/1s-suc	0.998374
Light1 vs. T1/4s-suc	0.321587
T2+L2 vs. Tone2	0.000001
T2+L2 vs. Light2	0.000084
T2+L2 vs. T1+L1	0.000005
T2+L2 vs. T1/2s-water	0.001518
T2+L2 vs. T1/1s-suc	0.942301
T2+L2 vs. T1/4s-suc	0.646258
Tone2 vs. Light2	0.701788
Tone2 vs. T1+L1	0.996313
Tone2 vs. T1/2s-water	0.169877
Tone2 vs. T1/1s-suc	0.000017
Tone2 vs. T1/4s-suc	0.000000
Light2 vs. T1+L1	0.986154
Light2 vs. T1/2s-water	0.986154
Light2 vs. T1/1s-suc	0.003249
Light2 vs. T1/4s-suc	0.000000
T1+L1 vs. T1/2s-water	0.589215
T1+L1 vs. T1/1s-suc	0.000187
T1+L1 vs. T1/4s-suc	0.000000
T1/2s-water vs. T1/1s-suc	0.043093
T1/2s-water vs. T1/4s-suc	0.000006
T1/1s-suc vs. T1/4s-suc	0.080265

Supplementary Table 5. Summary of post-hoc statistical comparisons (Bonferroni test) in Fig. 5B.

**Stimulus pairs**	**p-value**
Tone1 vs. Light1	0.999807824
Tone1 vs. T2+L2	1
Tone1 vs. Tone2	1.02E-07
Tone1 vs. Light2	9.06E-08
Tone1 vs. T1+L1	1.36E-07
Tone1 vs. T1/2s-water	2.08E-05
Tone1 vs. T1/1s-suc	0.398004802
Tone1 vs. T1/4s-suc	0.040178804
Light1 vs. T2+L2	0.999940341
Light1 vs. Tone2	4.55E-07
Light1 vs. Light2	1.23E-07
Light1 vs. T1+L1	1.32E-06
Light1 vs. Tone1/2s-water	0.000296036
Light1 vs. T1/1s-suc	0.767990654
Light1 vs. T1/4s-suc	0.006196083
T2+L2 vs. Tone2	1.10E-07
T2+L2 vs. Light2	9.12E-08
T2+L2 vs. T1+L1	1.65E-07
T2+L2 vs. T1/2s-water	3.10E-05
T2+L2 vs. T1/1s-suc	0.450253075
T2+L2 vs. T1/4s-suc	0.031485374
Tone2 vs. Light2	0.999985356
Tone2 vs. T1+L1	0.999999895
Tone2 vs. T1/2s-water	0.938758012
Tone2 vs. T1/1s-suc	0.001625976
Tone2 vs. T1/4s-suc	8.97E-08
Light2 vs. T1+L1	0.999620565
Light2 vs. T1/2s-water	0.767990654
Light2 vs. T1/1s-suc	0.000296036
Light2 vs. T1/4s-suc	8.97E-08
T1+L1 vs. T1/2s-water	0.978867399
T1+L1 vs. T1/1s-suc	0.003762377
T1+L1 vs. T1/4s-suc	8.97E-08
T1/2s-water vs. T1/1s-suc	0.120044926
T1/2s-water vs. T1/4s-suc	8.97E-08
T1/1s-suc vs. T1/4s-suc	2.64E-06

Supplementary Table 6. Summary of post-hoc statistical comparisons (Bonferroni test) Fig. 7A.

Stimulus pairs	p-values
T1/2s-water vs. T1/1s-suc	0.093779
T1/2s-water vs T1/2s-suc	0.000239
T1/2s-water vs. L1/2s-suc	0.000117
T1/2s-water vs. T2+L2/2s-suc	0.001158
T1/2s-water vs. T1/4s-suc	0.000000
T1/1s-suc vs. T1/2s-suc	0.536430
T1/1s-suc vs. L1/2s-suc	0.435188
T1/1s-suc vs. T2+L2/2s-suc	0.772107
T1/1s-suc vs. T1/4s-suc	0.000009
T1/2s-suc vs. L1/2s-suc	0.999988
T1/2s-suc vs. T2+L2/2s-suc	0.999106
T1/2s-suc vs. T1/4s-suc	0.012724
L1/2s-suc vs. T2+L2/2s-suc	0.995250
L1/2s-suc vs. T1/4s-suc	0.021051
T2+L2/2s-suc vs. T1/4s-suc	0.003410

Supplementary Table 7. Summary of simple regression analyses in individual neurons (F- and p-values) in Fig. 7D.

	Early CS^+-selective		Late CS^+-selective	
Neuron no.	F-value	p-value	F-value	p-value
1	F(1,5)=11.8346	0.0263	F(1,5)=52.3599	0.0019
2	F(1,5)=3.2545	0.1456	F(1,5)=42.5154	0.0029
3	F(1,5)=2.5634	0.1846	F(1,5)=11.5579	0.0273
4	F(1,5)=1.9189	0.2382	F(1,5)=10.9755	0.0296
5	F(1,5)=1.6763	0.2651	F(1,5)=9.9489	0.0344
6	F(1,5)=1.3938	0.3032	F(1,5)=9.4471	0.0372
7	F(1,5)=1.0409	0.3653	F(1,5)=8.5058	0.0434
8	F(1,5)=0.6075	0.4793	F(1,5)=8.4979	0.0435
9	F(1,5)=0.5721	0.4915	F(1,5)=7.8517	0.0487
10	F(1,5)=0.5488	0.4999	F(1,5)=4.7863	0.0939
11	F(1,5)=0.4858	0.5242	F(1,5)=3.4443	0.1371
12	F(1,5)=0.3524	0.5847	F(1,5)=2.7117	0.175
13	F(1,5)=0.3331	0.5948	F(1,5)=2.4093	0.1956
14	F(1,5)=0.1924	0.6836	F(1,5)=2.187	0.2133
15	F(1,5)=0.0829	0.7877	F(1,5)=2.0164	0.2286
16	F(1,5)=0.0474	0.8382	F(1,5)=1.8902	0.2412
17	F(1,5)=0.0412	0.8491	F(1,5)=1.8059	0.2502
18	F(1,5)=0.0325	0.8656	F(1,5)=0.4554	0.5368
19	F(1,5)=0.0097	0.9262	F(1,5)=0.3481	0.5869
20	F(1,5)=0.0055	0.9444	F(1,5)=0.0318	0.8672
